# Fibroblast Growth Factor Receptors (FGFRs) and Noncanonical Partners in Cancer Signaling

**DOI:** 10.3390/cells10051201

**Published:** 2021-05-14

**Authors:** Harriet R. Ferguson, Michael P. Smith, Chiara Francavilla

**Affiliations:** 1 Division of Molecular and Cellular Function, School of Biological Science, Faculty of Biology Medicine and Health (FBMH), The University of Manchester, Manchester M13 9PT, UK; harriet.ferguson-2@postgrad.manchester.ac.uk; 2 Manchester Breast Centre, Manchester Cancer Research Centre, The University of Manchester, Manchester M20 4GJ, UK

**Keywords:** FGFRs, FGFs, signaling, cancer, tumorigenesis, coreceptors, cell adhesion molecules, extracellular matrix, receptor tyrosine kinase, EGFR

## Abstract

Increasing evidence indicates that success of targeted therapies in the treatment of cancer is context-dependent and is influenced by a complex crosstalk between signaling pathways and between cell types in the tumor. The Fibroblast Growth Factor (FGF)/FGF receptor (FGFR) signaling axis highlights the importance of such context-dependent signaling in cancer. Aberrant FGFR signaling has been characterized in almost all cancer types, most commonly non-small cell lung cancer (NSCLC), breast cancer, glioblastoma, prostate cancer and gastrointestinal cancer. This occurs primarily through amplification and over-expression of *FGFR1* and *FGFR2* resulting in ligand-independent activation. Mutations and translocations of *FGFR1-4* are also identified in cancer. Canonical FGF-FGFR signaling is tightly regulated by ligand-receptor combinations as well as direct interactions with the FGFR coreceptors heparan sulfate proteoglycans (HSPGs) and Klotho. Noncanonical FGFR signaling partners have been implicated in differential regulation of FGFR signaling. FGFR directly interacts with cell adhesion molecules (CAMs) and extracellular matrix (ECM) proteins, contributing to invasive and migratory properties of cancer cells, whereas interactions with other receptor tyrosine kinases (RTKs) regulate angiogenic, resistance to therapy, and metastatic potential of cancer cells. The diversity in FGFR signaling partners supports a role for FGFR signaling in cancer, independent of genetic aberration.

## 1. Introduction

The superfamily of Receptor Tyrosine Kinases (RTKs) comprises 20 subfamilies of cell-surface receptors with conserved structures. Upon activation, RTKs undergo dimerization, internalization and initiate large-scale tyrosine phosphorylation responses and signaling cascades to regulate cell growth, proliferation, survival and differentiation [1]. The importance of RTKs in cancer was established with the successful introduction of Gleevec, Herceptin and Iressa, the first RTK inhibitors to show antitumor effects approved for clinical use in the early 2000s [2]. In tandem, the role of RTKs in relation to development, tissue homeostasis and other diseases was a growing body of research.

Among RTKs, the family of Fibroblast Growth Factor Receptors (FGFRs) comprises four genes that give rise to at least seven different receptor isoforms. These receptors are differentially activated by one of the 22 Fibroblast Growth Factor (FGF) ligands with known FGFR-binding activity, which are nearly ubiquitously expressed in all adult tissues and play a critical role in development, tissue homeostasis and human diseases [3]. Like other RTKs, *FGFR1-4* have been implicated in cancers arising from nearly all tissue types [4]. In-line with the number of ligands and receptor variants, FGFR signaling is highly context-specific, which is evidenced by its tumorigenic or tumor suppressor roles in different cancer types. For example, *FGFR2* loss of function mutations have been reported in 10% of melanoma tumors and cell lines [5] as well as in bladder cancers. *FGFR2* downregulation has also been reported in bladder cancer [6] and in subtypes of hepatocellular carcinoma (HCC) [7], whereas in other cancer types/subtypes with overexpression of the *FGFR2*, including gastric cancer [8,9], triple negative breast cancer [10] and osteosarcoma [11], inhibition of the receptor decreases cancer cell proliferation and cell survival in vitro. Beyond canonical FGFR signaling and signaling partners, the FGFR interactome is large, and how noncanonical interaction partners regulate FGFR signaling in tumorigenesis is not fully understood [12,13]. Cell surface FGFR interactions with extracellular matrix (ECM) proteins, cell adhesion molecules (CAMs), coreceptors and other RTKs are known to be critical during development [14]. Such heterotypic interactors of FGFR also have a role in tumorigenesis and tumor progression. Here, we outline the current understanding of the role of heterotypic FGFR interactors in regulating FGFR signaling in cancer using selected examples to illustrate each concept. First, we will focus on the role of canonical FGFR signaling partners, such as heparan sulfate proteoglycans (HSPGs) and Klotho, in FGFR signaling. Finally, we discuss noncanonical signaling partners of FGFR for which a role in cancer has been identified.

## 2. The FGFR Family

**Figure 1:**
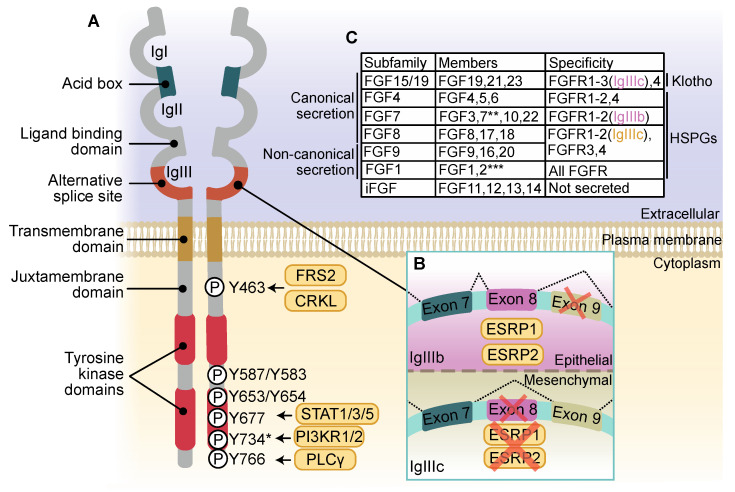
Fibroblast Growth Factor Receptor (FGFR) structure, activation and Fibroblast Growth Factor (FGF)-FGFR specificity. (**A**) The extracellular domain of FGFR comprises three immunoglobulin-like looped domains (IgI-IgIII) with an acid box that sits between IgI and IgII. FGF binds the receptor between IgII and IgIII. A long juxtamembrane domain contains the binding site for FGFR substrate 2 (FRS2) and chicken tumor virus no. 10 regulator of kinase (CRK) or the closely related CRK-like (CRKL). Across two tyrosine kinase domains, phosphorylated tyrosine residues are required for full activation of the receptor and docking of signal transducer and activator of transcription 1/3/5 (STAT1/3/5), p85 subunit of phosphatidylinositide 3-kinase subunit α/β (PI3KR1/2) and phospholipase C gamma (PLCγ). Within IgIII lies an alternative splice site that gives rise to FGFR IgIIIb and IgIIIc isoforms. (**B**) Epithelial splicing regulatory proteins 1 (ESRP1) and ESRP2 present in epithelial cells regulates inclusion of exon 8 and exclusion of exon 9 to give rise to FGFR-IgIIIb isoform. The absence of ESRP1/2 in mesenchymal cells results in inclusion of exon 9 and exclusion of exon 8 giving rise to IgIIIc isoform. Alternative splicing gives rise to FGFR isoforms with different ligand binding affinities. (**C**) FGF subfamilies have different FGFR-binding affinities and mechanisms of secretion, or in the case of intracellular FGFs (iFGF) are not secreted. FGFR requires different coreceptors Klotho or heparan sulfate proteoglycan (HSPG) to activate different repertoire of FGFR isoform. * Y734 corresponds to FGFR2-IgIIIb only; ** can bind FGFR2-IgIIIb isoform only; *** cannot bind FGFR2-IgIIIb isoform.

FGFR was first described as a receptor for basic fibroblast growth factor (bFGF, later termed FGF2) purified from chicken embryos with an extracellular domain containing three immunoglobulin-like looped domains (Ig domains) and an acidic region (known as the acid box), a single transmembrane domain, a long juxtamembrane domain and an intracellular domain with two tyrosine kinase domains (Figure 1A) [15]. Previously identified *FLG* in human cDNA screens [16], now known as *FGFR1*, was shown to respond to acidic FGF (aFGF, then renamed FGF1) [17]. *FGFR2*, originally *BEK*, was identified as sharing tyrosine-kinase similarity to *FGFR1* [18], leading to the discovery of RTKs encoded by the genes *FGFR3* and *FGFR4* with highly conserved structures to *FGFR1* and *FGFR2* [19,20]. FGFR5, also known as FGFR like 1 (FGFRL1), is considered a fifth member of the FGFR family. Two isoforms isolated from cDNA libraries, FGFR5β and FGFR5γ, have three and two Ig domains, respectively, differentiating them from the other FGFR family members. Both FGFR5 isoforms lack a tyrosine kinase domain, and as such were traditionally considered negative regulators of FGFR1-4 [21,22]. Characterization of FGFR5-regulated signaling has revealed that FGFR5 can act as a coreceptor to enhance FGFR1 signaling [23,24].

In the third Ig domain (IgIII) of the extracellular domain of FGFR1–3, alternative splicing of three exons gives rise to two different isoforms, FGFR(1–3)-IgIIIb and -IgIIIc (Figure 1B) [25]. The alternatively spliced FGFR isoforms have different ligand-binding affinities (Figure 1C) [26,27]. For instance, the binding of FGFR2-IgIIIb-specific keratinocyte growth factor (KGF, known as FGF7) to FGFR2-IgIIIc can be induced by introducing an FGFR2b-specific IgIII variable region [28]. The IgIII isoforms of FGFR show tissue and developmental stage-specific expression patterns [29]. The IgIIIb variant is preferentially expressed in epithelial tissues, which is regulated by activity of epithelial splicing regulatory protein 1 (ESRP1) and ESRP2 (Figure 1B). Overexpression of ESRP1 and ESRP2 in mesenchymal cells is sufficient to switch expression from the FGFR2-IgIIIc to the FGFR2-IgIIIb isoform [30]. Alternative splicing of the extracellular domain of FGFR1 also gives rise to an FGFR1α type 1 (FGFR1α2), FGFR1α2, FGFR1β and FGFR1γ isoform of the receptor. FGFR1α1/2 have three Ig domains, whereas FGFR1β and FGFR1γ lack the N-terminal IgI domain [31,32,33]. FGFR1γ differs from FGFR1β in that alternative splicing also skips the exon containing the acid box. This IgI/acid box skipping regulates ligand-binding affinities of the FGFR1 isoforms [33,34]. FGFR1α1 has a classical FGFR1 structure. FGFR1α2, however, has a truncated C-terminal kinase domain, subsequently lacking critical tyrosine autophosphorylation sites. As a result, FGFR1α2 is defective in initiating canonical FGFR1 signaling cascades. An FGFR2β isoform, and an FGFR3 isoform lacking the acid box designated FGFR3ΔAB, have also been identified [35,36,37].

## 3. FGFR Activation and Signaling

**Figure 2:**
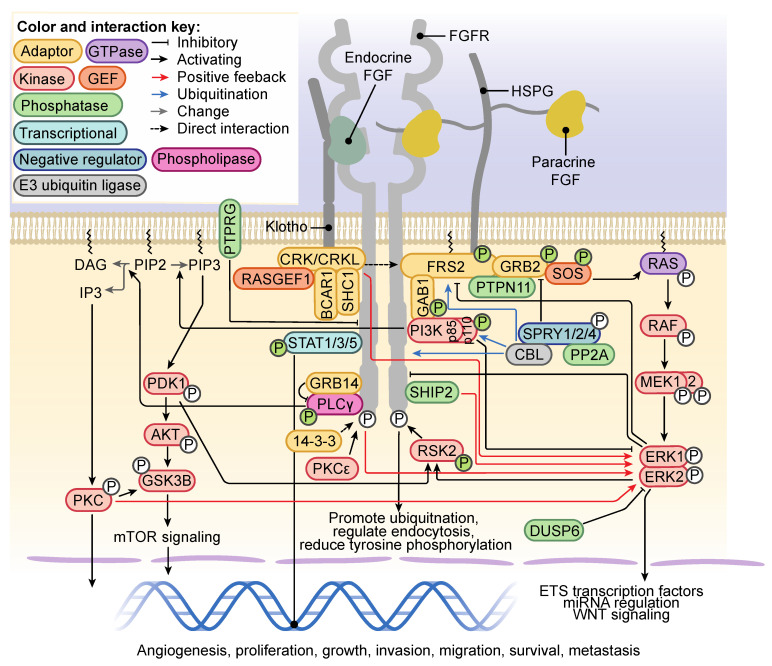
Fibroblast Growth Factor Receptor (FGFR) signaling partners, pathways and regulation of FGFR signaling Klotho or heparan sulfate proteoglycan (HSPG) are required for activation of FGFR by endocrine or paracrine FGFs, respectively. Binding of the ligand initiates large-scale phosphorylation and activation of intracellular signaling cascades. RAS-mitogen activated protein kinase (MAPK) and phosphatidylinositide 3-kinase (PI3K)-AKT signaling are dependent on FRS2 binding FGFR, which is associated with a CRK/CRKL complex to positively regulate extracellular signal-regulated protein kinase 1/2 (ERK1/2) signaling. ERK1/2 signaling is tightly regulated downstream of FGFR. The PI3K p85 subunit can bind FGFR independently of growth factor receptor bound 2 (GRB2)-associated binding protein 1 (GAB1) to activate AKT signaling through conversion of phosphatidylinositol 4,5-bisphosphate (PIP_2_) to phosphatidylinositol 3,4,5-trisphosphate (PIP_3_). PI3K-AKT signaling activates the mammalian target of rapamycin (mTOR) signaling downstream FGFR activation. PLCγ binds FGFR and hydrolyses PIP_2_ to produce diacylglycerol (DAG) and inositol trisphosphate (IP_3_). IP_3_ activates protein kinase C (PKC) signaling. STAT1/3/5 activation by FGFR results in translocation to the nucleus to regulate transcription. Positive regulators of FGFR signaling include 14-3-3, protein kinase C ε (PKCε) and PIP_3_ 5-phosphatase 2 (SHIP2), whereas negative regulators include SPROUTY 1/2/4 (SPRY1/2/4), E3-ubiquitin ligase CBL (CBL), protein phosphatase 2 A (PP2A), dual specificity phosphatase 6 (DUSP6) and protein tyrosine phosphatase receptor type G (PTPRG). Phosphorylation colored green indicates proteins where activation is dependent on FGFR-mediated tyrosine phosphorylation.

Following binding of FGF, FGFRs predominantly form an asymmetric homodimer with the first kinase domain of one molecule interacting with the second kinase domain of its dimerization partner to facilitate transautophosphorylation [38]. Dimerization of the transmembrane domain is negatively regulated by a conserved motif within the C-terminal juxtamembrane domain, likely preventing ligand-independent dimerization [39]. Key signaling events are summarized in Figure 2. Autophosphorylation of specific residues within the intracellular domain recruits specific signaling adaptors to activate different signaling nodules (Figure 1A). Tyrosine (Y) 653 and Y654 of FGFR1 are required for kinase activation and consequent RAS/mitogen activated protein kinase (MAPK) signaling [40], whereas specific autophosphorylation of Y766 is required for docking of the SRC homology 2 (SH2)-domain of phospholipase C gamma (PLCγ) and subsequent PLCγ activation [41]. The p85 phosphatidylinositide 3-kinase subunit α/β (PI3KR1/2) is recruited to Y734 of FGFR2-IgIIIb in response to FGF10 treatment [42], as is seen for Y760 phosphorylation of FGFR3 in response to FGF2 [43]. Crucial to FGFR-RAS-MAPK signaling is the activation of FGFR substrate 2 (FRS2), a lipid-anchored adaptor protein constitutively bound to the juxtamembrane of FGFR [44,45]. FGFR-mediated phosphorylation of Y346 of FRS2 functions as a docking site for the N-terminal SH2 domain of protein tyrosine phosphatase nonreceptor type 11 (PTPN11, also known as SHP2) [46]. Growth factor receptor-bound 2 (GRB2) is indirectly recruited by PTPN11, and independently downstream of Y196, Y306, Y349 and Y392 FRS2 phosphorylation (Figure 2) [44]. FRS2 recruitment sustains RAS-MAPK signaling in an FGFR-specific manner, in comparison to pan-RTK RAS-MAPK adaptor SH2-containing (SHC) transforming protein 1 (SHC1) [46]. A second adaptor that directly interacts with FGFR is the chicken tumor virus no. 10 regulator of kinase (CRK) and the closely related CRK-like (CRKL). The CRK/CRKL SH2-domain directly binds phosphorylated Y463-FGFR1 [47,48] and can interact with FRS2-GRB2-PTPN11 [49] in a complex containing SHC, breast cancer anti-estrogen resistance protein 1 (BCAR1) and RAP guanine nucleotide exchange factor 1 (RAPGEF1) (Figure 2) [47] to regulate efficient activation of extracellular signal-regulated protein kinase 1/2 (ERK1/2) of the RAS-MAPK pathway [48,50]. ERK1/2 signal duration can be regulated by phosphatidylinositol 3,4,5-trisphosphate 5-phosphatase 2 (SHIP2), an adaptor of FGFR signaling. SHIP2 interactions with FGFR to mediate a sustained ERK1/2 signal, which is transient in the absence of SHIP2. This is achieved through SHIP2-regulation of FRS2 and recruitment of SRC, proto-oncogene, non-receptor tyrosine kinase (SRC)-family kinases (SFK) [51]. ERK1/2 directly phosphorylates low-density lipoprotein receptor-related protein 6 (LRP6) at S1490 and T1572 downstream of FGFR2/3, which increases WNT signaling [52]. Ribosomal S6 kinase 2 (RSK2), a serine/threonine kinase activated by 3-phosphoinositide-dependent protein kinase 1 (PDK1) and ERK1/2 phosphorylation, is directly phosphorylated by FGFR3 at sites Y529 and Y707. Respectively, these sites facilitate binding of inactive ERK1/2 and remove autoinhibition of RSK2 (Figure 2) [53,54]. GRB2-associated binding protein 1 (GAB1) is recruited to phosphorylated FRS2, where it is phosphorylated and also recruits PI3K to activate PI3K-AKT signaling [55]. PI3K-AKT signaling downstream of FGFR activation is involved in FGF-mediated angiogenesis [56,57] and negatively regulates ERK1/2 signaling [43]. FGFR1-4 signaling can activate signal transducer and activator of transcription 1 (STAT1), STAT3 and STAT5 signaling (Figure 2) [58,59,60,61]. STAT3 binding and subsequent activation are dependent on phosphorylation of a receptor-isoform conserved Y677 (FGFR1) and FGF-activated SRC and non-receptor tyrosine kinase Janus kinase (JAK) [62].

## 4. Regulation of FGFR Signaling and FGFR Trafficking

Mechanisms exist to amplify FGFR-dependent signaling (Figure 2), for example, protein kinase C ε (PKCε) phosphorylates S779 of FGFR1 and FGFR2, a docking site for the adaptor 14-3-3, which binds and amplifies FGFR-RAS-MAPK and FGFR-PI3K-AKT signaling [63,64]. Alternatively, negative feedback mechanisms prevent aberrant FGFR signaling implicated in multiple pathologies including cancer. Active ERK1/2 directly phosphorylates a conserved S777 of FGFR1, not dependent on FGFR activation, which when inhibited enhances FGFR signaling [65]. FGFR-dependent activation of ERK1/2 also phosphorylates FRS2 at eight threonine residues to reduce FGFR-FRS2 signaling [66], which in the absence of ERK1/2 can be mediated by p38 MAPK [67]. Activated RSK2, a downstream target of ERK1/2 signaling, binds and phosphorylates S789 of FGFR1 to reduce tyrosine phosphorylation, promote ubiquitination and regulate endocytosis of the receptor [63].

MicroRNAs (miRNAs), small noncoding RNAs, classically 20–22 nucleotides long, can regulate gene expression by repression of transcription and translation [68]. FGFR1 increases expression of miR-214-3p in an ERK1/2-activation-dependent manner, which subsequently downregulates expression of FGFR1 [69]. A complex feedback loop also exists between the miR-15/16 subfamily of miRNAs and FGF/FGFR signaling. FGFR activation negatively regulates expression of miR-15/16 subfamily members miR-15a, miR-15b and miR-16. This reduces miR-15/16-mediated transcriptional-repression of both FGFs and FGFRs [70]. miRNA downregulation of FGFR expression has been studied in relation to cancer progression but not necessarily as part of a feedback mechanism of FGFR activation. Expression of FGFR1 targeting miR-214 is decreased in a subset of HCCs, which is associated with increased FGFR1 expression [71]. Other examples include miR-133b direct downregulation of FGFR1 expression and RAS-MAPK/PI3K-AKT signaling in osteosarcoma cells [72], miR-889-3p decreasing FGFR2 expression in cervical cancer [73] and miR-24-3p downregulation of FGFR3 expression in multiple myeloma (MM) and lung adenocarcinoma [74].

SPROUTY (SPRY) proteins are also negative regulators of RTK signaling. SPRY1/2 dissociates from GRB2 following PTPN11-mediated dephosphorylation, facilitating the GRB2-FRS2 interaction downstream of FGFR activation. SPRY1/2/4 are considered tumor suppressors for their negative regulation of RAS-MAPK activity [75,76]. SPRY Y55 phosphorylation is required for SPRY interaction with E3-ubiqutin ligase CBL and serine/threonine protein phosphatase 2A (PP2A) (Figure 2), and inhibition of RAS-MAPK signaling [77,78,79,80]. These SPRY actions are dependent on FRS2 and SFK activity downstream of FGF2 [81,82]. Other negative regulators of FGFR signaling include protein tyrosine phosphatase receptor type G (PTPRG), which colocalizes with and suppresses FGFR1 activity at the plasma membrane [83], and dual specificity phosphatase 6 (DUSP6, often referred to as MKP3) which represses FGFR activity [84].

Growth-factor receptor bound 14 (GRB14) is recruited to the PLCγ binding site; the phosphorylated Y766 of FGFR1. As an FGFR1-PLCγ-GRB14 complex, GRB14 inhibits PLCγ phosphorylation by FGFR1 and subsequent downstream PLCγ-signaling events (Figure 2) [85,86,87]. The activity of GRB14 bound to FGFR1 has also been shown to block activation of ERK1/2, MAPK8 (also known as JNK1) and AKT signaling [86].

The E3-ubiquitin ligase CBL recognizes consensus sequences containing tyrosine phosphorylation to bind target proteins. Following binding, CBL catalyzes mono and polyubiquitination of the target protein, which is predominantly associated with promoting either lysosomal or proteasomal degradation [88]. CBL is recruited to the FGFR-FRS2-GRB2 complex, where it binds GRB2 and ubiquitinates FRS2 and FGFR (Figure 2) [89]. Interactions with PI3K increase following FGFR2 activation, which attenuates PI3K-AKT signaling in osteoblasts [90]. Ubiquitination of FGFR following activation is required for sorting of the receptor into the lysosome for degradation to regulate FGFR signal duration. Mutation of lysine residues within the intracellular domain of FGFR to prevent ubiquitination promotes sorting of FGFR into the recycling endosome [91].

Ligand-dependent signaling is, in part, regulated by differential trafficking of the receptor isoforms. FGF1 activation of FGFR1-3 IgIIIc isoforms causes rapid sorting of the receptors into late endosomes/lysosomes, most rapidly FGFR1-IgIIIc, while FGFR4 is sorted to the recycling compartment [92]. Similarly, FGF7 induces degradation of FGFR2-IgIIIb through recruitment of CBL and increased ubiquitination of the receptor, giving rise to transient ERK1/2 activation. Alternatively, FGF10-mediated autophosphorylation of Y734 recruits PI3KR1/2 and SH3 binding protein 4 (SH3BP4), which instead results in sorting to the recycling compartment, sustained ERK1/2 activation and higher mitogenic activity [42,93].

In conclusion, FGF/FGFR signaling requires tight regulation through different mechanisms, and this regulation is often lost in cancer.

## 5. FGF Signaling Diversity in Cancer

Seven families of FGF have been characterized based on expression, receptor-specificity and downstream signaling axis similarities. A total of 22 *FGF* genes gives rise to seven subfamilies: FGF1, FGF4, FGF7, FGF8, FGF9, FGF15/19 and intracellular FGF (iFGF) [3] (Figure 1C). FGF oncogenic signaling primarily occurs through *FGFR1-4* amplification, translocation and/or increased expression, and very rarely have mutations in *FGF* genes been associated with oncogenic signaling [3]. Despite this, autocrine, paracrine and endocrine FGF signaling have been implicated in cancer [94]. In the following sections we discuss the diversity of roles of FGF subfamilies and their receptors in several cancer types through illustrative examples (Table 1, Table 2), highlighting intrinsic differences in FGFR biology that contribute to a complicated network of FGF-FGFR signaling in cancer.

### 5.1. The FGF1 Subfamily

The FGF1 subfamily includes secreted ligands FGF1 and FGF2, which both lack an N-terminal classical-secretion signal peptide [126]. These alternative routes of secretion are considered a mechanism of preventing aberrant FGF1 and FGF2 signaling [127]. The FGF1 subfamily, as well as the FGF4, FGF7, FGF8 and FGF9 subfamilies, require the presence of the coreceptor HSPG, a transmembrane proteoglycan that binds FGFs and stabilizes FGF-FGFR interactions to regulate downstream signaling events [128,129,130,131,132]. FGF1 and FGF2 have promiscuity in receptor specificity in common; however, only FGF1 has been shown to bind all receptor isoforms (Figure 1C) [3]. With respect to genomic aberrations in the FGF1 subfamily, a study investigating SNPs and risk of breast cancer found that, on their own, SNPs in *FGF1* and *FGF2* were not associated with increased risk, but an *FGF1* SNP was associated with decreased survival [133]. *FGF1* amplification in ovarian cancer has been associated with promoting angiogenesis, reduced disease-free progression and overall survival [134,135]. FGF1 expression in ovarian cancer is associated with chemotherapy resistance. FGF1 can reduce the transcriptional activity of p53 and increase expression of p21 and subsequent antiapoptotic activity in response to ovarian cancer therapies etoposide and cisplatin [98]. FGF1 expression and secretion are increased in ovarian cancer associated fibroblasts (CAFs), which, when cultured with the SKOV3 human ovarian cancer cell line, increases ERK1/2 activity through activation of FGFR4. This activity increased expression of all markers for epithelial mesenchymal transition (EMT), the process of cellular reprogramming whereby epithelial cells remove cell-cell and basement membrane contacts, and transition to a mesenchymal phenotype [136]. EMT is considered crucial for cancer cell invasion, migration and metastasis [137]. Similarly, CAF secretion of FGF2 in breast cancer can bypass classical hormone receptor signaling inhibited by endocrine therapy to activate ERK1/2 signaling, promote MYC target gene expression and tumor growth. This suggests FGF2 secretion by CAFs has a role in endocrine therapy resistance in hormone receptor-positive breast cancer [138,139,140]. FGF2 is also overexpressed in subtypes of bladder cancer, and is associated with promoting migration, angiogenesis and invasiveness [141,142]. FGF2 expression by tumor vasculature pericytes has also been identified as a mechanism of anti-vascular endothelial growth factor receptor (VEGFR) resistance [143]. FGF2 is an angiogenic factor in multiple myeloma (MM) that positively regulates expression of growth and survival interleukin-6 (IL-6) in a reciprocal manner [144]. The use of FGF traps as inhibitors of FGF/FGFR signaling in MM cells has shown the FGF/FGFR signaling axis is required for stabilization of the oncoprotein, transcription factor c-MYC and subsequent resistance to mitochondrial oxidative stress induced apoptosis, which is critical in tumor progression [145,146]. Nuclear activity of a heavy molecular weight FGF2 (hmwFGF2) [147] has been implicated in promoting cell proliferation and survival in glioma [148,149], in glioma cells, and indirectly through nuclear localization in astrocytes [149]. It is likely that nuclear hmwFGF2 induces this effect partly through decreased expression of tumor suppressor phosphatase and tensin homolog (PTEN), as well as increased AKT activity [148]. Low molecular weight (lmw) FGF2-FGFR1 colocalization in the nucleus, and subsequent activity in pancreatic stellate cells (PSCs), promotes proliferation and PSC-mediated pancreatic cancer cell invasion [150]. Recently a pro-survival role for nuclear lmwFGF2 has been characterized, whereby FGF2 colocalizes in the nucleus with apoptosis inhibitor 5 (API5) to promote mRNA export of cyclin D1 and c-MYC [151].

### 5.2. The FGF4 Subfamily

The FGF4 subfamily contains FGF4, FGF5 and FGF6, which have common receptor-binding affinities, binding the mesenchymal FGFR1-3-IgIIIc isoforms as well as FGFR4 (Figure 1C) [152]. An N-terminal signal peptide regulates secretion of the FGF4 subfamily through ER-Golgi entry [153]. Amplification of the 11q13 amplicon, present in breast cancer, results in co-amplification of *FGF3*, *FGF4* and *FGF19* [154], but is also observed in other cancer types [155]. In the presence of CAFs, expression of FGF4 and FGFR2 is increased in cancer stem cells isolated from the ovarian cancer cell line HTBoA. Cancer stem cells are commonly referred to as cancer initiating cells for their tumorigenic activity associated with resistance to therapy, increased recurrence and metastasis. FGF4 expression in these cancer stem cells increased sphere-formation, which could be reduced by silencing of FGFR2 [156]. In molecular subtypes of gastrointestinal stromal tumors (GISTs), which lack any of four common biomarkers referred to as *quadruple* WT GIST, *FGF4* amplification and increased expression resulted in increased AKT activation downstream of FGFR1, and has been suggested as a therapeutic target in this molecular subtype [102].

### 5.3. The FGF7 Subfamily

The FGF7 subfamily, which comprises FGF3, FGF7, FGF10 and FGF22, is expressed in mesenchymal cells and shows high affinity for epithelial FGFR2-IgIIIb binding, less so for FGFR1-IgIIIb, and none for FGFR3-IgIIIb [152,157]. This high-specificity of FGF7 subfamily members is partly underpinned by hydrogen bonds, dependent on the unique aspartic acid residue D76 present in FGF7, but not FGF10, within an interaction site with serine 315 of FGFR2-IgIIIb [158]. Despite this unanimous subfamily specificity, gene knockout studies show that the roles of these four family members are nonredundant [159]. In human gastric cancer cells, FGF7-FGFR2 signaling increased PI3K-AKT-mTOR signaling, which upregulated invasive/migratory glycoprotein thrombospondin 1 (THBS1). This FGF7-FGFR2-THBS1 activity increases migration and invasion of gastric cancer cells [105]. FGF10-FGFR2b signaling promotes migration of breast cancer and pancreatic adenocarcinoma (PDAC) cells [42,108,160]. FGF10 is expressed in PDAC stromal cells, while FGFR2 is expressed in cancer cells and is associated with poor prognosis [108]

### 5.4. The FGF8 Subfamily

FGF8, FGF17 and FGF18 belong to the secreted FGF8 subfamily, which shows the highest affinity for the FGFR3-IgIIIc isoform and an FGFR4 isoform with two Ig-domains [157]. A property of FGF8 subfamily ligands is regulation of isoform expression by alternative splicing. Alternative splicing of *FGF8* gives rise to four isoforms, FGF8a/b/e/f, which have divergent roles in development [161]. FGF8 expression has been linked to neoadjuvant radiochemotherapy resistance in colorectal cancer (CRC). The level of FGF8 detected by immunohistochemistry was increased in nonresponsive patient samples in comparison to responsive patients. This was associated with increased expression of survivin (*BIRC5*), which is an inhibitor of apoptosis. Exposure of CRC cell lines to radiation increased expression of FGF8 and FGFR3 in the surviving cell population [109].

### 5.5. FGF9 Subfamily

Like the FGF1 subfamily, FGF9, FGF16 and FGF20, which make up the FGF9 subfamily, do not have an N-terminal signal peptide implicated in ER-Golgi transport. Despite this, FGF9 is secreted via ER-Golgi-mediated transport [162,163,164] using an alternative secretion signal dependent on two hydrophobic regions located centrally and N-terminally [165,166]. FGF9 has been implicated in crosstalk between HCC cells and hepatic stellate cells, which promotes liver fibrosis and tumor progression. FGF9 is expressed by hepatic stellate cells, which signals to HCC cells in a paracrine manner to activate ERK1/2 and JNK signaling, promote migration and proliferation. Furthermore, stimulation of HCC cells with FGF9 reduced sensitivity to the HCC therapy sorafenib [115].

### 5.6. FGF15/19 Subfamily

The FGF15/19 subfamily, commonly referred to as the endocrine FGFs, consists of FGF19 (human ortholog of mouse FGF15), FGF21 and FGF23, which have an endocrine function regulated by the FGFR coreceptor Klotho [3]. Klotho directly interacts with a conserved C-terminal sequence of FGF15/19 subfamily ligands to coordinate binding with FGFR and to activate signaling [167]. Different Klotho species regulate the affinity of FGFRs for FGF ligands. For example, Klotho-β (*KLB*) is required for FGF21 activation of FGFR1 and FGFR3-IgIIIc isoforms, whereas FGF23 requires Klotho-α (*KL*) [168]. Genomic aberrations in the *FGF19* subfamily members have rarely been associated with a role in cancer progression, with the exception of an SNP in *FGF23* that has been linked to increased risk of prostate cancer [169]. Although not directly linked to the SNP, FGF23 overexpression has also been associated with reduced overall survival and shortened time to occurrence of bone metastases [170] A large proportion of endometrial cancers are associated with metabolic disorders, including obesity. FGF21 and FGF23 expression have been implicated in endometrial cancer due to their association with increased expression of leptin (LEP), a hormone produced in adipose tissue. Serum FGF21 and LEP concentrations are increased in patients with endometrial cancer, and associated with low differentiation and higher grade of tumor [119]. Circulating FGF21 has also been associated with increased risk of CRC [118].

### 5.7. Intracellular FGFs

The intracellular FGFs (iFGFs), or fibroblast homologous factors (FHFs), family includes FGF11-14 (or FHF1-4), a subfamily of FGFs with no signal sequence that are not secreted and instead are considered cytosolic growth factors [171]. Traditionally, this subfamily has been considered to function independently of FGFR, instead binding to islet brain-2 (IB2) and sodium channels [172,173]. However, recent studies have shown that exogenous iFGFs can activate signaling. Treatment of cells with FGF11 and FGF13 can activate signaling cascades [174,175] and, in the case of FGF11, can initiate antiapoptotic processes and bind all four FGFRs to activate signaling when ectopically expressed [174]. Given that a mechanism of iFGF secretion has not been described, the physiological role of iFGFs in activating FGFR remains to be elucidated. Despite this, iFGFs have known functions in promoting tumor progression. FGF11-miR-541 activity in response to T-cell infiltration in prostate cancer contributes to prostate cancer cell invasion, likely through suppression of androgen receptor and matrix metallopeptidase 9 (MMP9) activity [123]. A recent study focused on the role of FGF13 expression in triple negative breast cancer (TNBC) (ER-, progesterone receptor-, and human epidermal growth factor receptor 2 (HER2/ERBB2)-negative breast cancer), an aggressive subtype of breast cancer where FGF13 expression was associated with relapse but was not increased in primary breast tumors. In a highly metastatic variant of human TNBC cell line MDA-MB-231, suppression of FGF13 removed almost all ability to metastasize to liver and lung, suggesting FGF13 has a role in determining a subset of TNBC’s propensity for metastasis to specific sites [125]. FGF14 expression, on the other hand, has been associated with tumor suppressor activity, which is downregulated in CRC tumor samples and in CRC cell lines as a result of high methylation of *FGF14*. Restoring FGF14 expression significantly reduces CRC tumor growth in vivo, associated with downregulation of PI3K-AKT-mTOR signaling in vitro [122].

## 6. FGFR Genomic Aberrations in Cancer

Perturbed FGFR signaling has been implicated across cancer subtypes in both cancer development and progression. Mechanisms of *FGFR1-4*-mediated oncogenic signaling include amplifications and/or overexpression, missense mutations, translocations and, in some cancer subtypes, loss of *FGFR1-4* expression. Cancer types with known *FGFR1-4* aberrations are summarized in Table 2.

**Table 2:** Examples of cancer subtypes for which Fibroblast Growth Factor Receptor (FGFR) genetic aberrations have been identified.

FGFR Gene	Cancer Subtype	Associated References
FGFR1	Breast cancer	[10,176,177]
	Colorectal cancer	[178,179]
	Gastrointestinal	[180]
	Glioma	[181,182]
	Head and neck	[183]
	Non-small cell lung cancer	[184,185,186,187,188]
	Ovarian cancer	[189]
	Pancreatic ductal adenocarcinoma	[190]
	Prostate cancer	[191,192,193,194]
	Small cell lung cancer	[186,195,196,197]
	Urothelial cancer	[189]
FGFR2	Breast cancer	[10,198]
	Cholangiocarcinoma	[199,200,201]
	Endometrial cancer	[202]
	Gastric cancer	[9,203,204]
	Non-small cell lung cancer	[205]
FGFR3	Bladder cancer	[189,206,207,208,209,210,211,212,213,214]
	Cervical cancer	[209,215,216]
	Glioma	[181,189]
	Non-small cell lung cancer	[189,205,217,218]
FGFR4	Adrenocortical cancers	[219]
	Breast cancer	[189]

### 6.1. FGFR1

Amplifications are the most common *FGFR1-4* genomic aberrations. Analysis of 4873 cancers found 61% of *FGFR1-4* aberrations (7.1% of all cancers) were amplifications. *FGFR1* amplifications accounted for approximately 41% of all *FGFR1-4* genomic aberrations [189]. Amplifications in *FGFR1* (8p11) are most commonly present in squamous cell carcinoma [184], more common than other non-small cell carcinomas and associated with late-stage [185], but not necessarily with poor prognosis [220,221]. In non-small cell lung cancer (NSCLC), *FGFR1* amplification is detected at a rate of 6% (*n* = 63) [186]. Inhibition of FGF/FGFR1 signaling in NSCLC has shown that the FGF/FGFR1 signaling axis promotes survival and resistance to oxidative stress in an MYC-dependent manner [222]. Analysis of 1875 breast tumor samples showed that *FGFR1* 8p11-12 amplification occurs in 10.5% of breast cancers, associated with estrogen receptor (ER) expression and lobular breast cancer [176], and in 8.9% of a cohort of 595 breast cancer tumors [177]. *FGFR1* amplifications in breast cancer are associated with lower rates of five-year disease-free survival, overall-survival and resistance to endocrine therapy [176,223,224,225]. Genetic analysis of 38 paired hormone sensitive and post-hormone resistant tumors found that 47% of all tumors had amplification of *FGFR1* or *FGFR2* (most common). None of the pre-hormone-sensitive tumors had *FGFR1* amplification; however, 17.65% of the post-hormone-resistant tumors had *FGFR1* amplification [226]. Moreover, *FGFR1* amplification has been implicated in resistance to hormone receptor therapies [223]. Other cancers with amplification of *FGFR1* genes include *FGFR1* in pancreatic adenocarcinoma (PDAC) [190], likely expressing the FGFR1-IgIIIc isoform [227,228], with *FGFR1* amplification in ovarian and urothelial cancer [189].

### 6.2. FGFR2

*FGFR2* amplifications are the second most common amplifications of *FGFR1-4* in cancer [189]. Interestingly, in the aggressive TNBC subtype, only *FGFR2* amplification (10q26) and overexpression are detected, occurring in 4% of tumors [10]. *FGFR2* amplification is also prominent in gastric cancer. A large multicenter study found *FGFR2* amplification in 7.4, 4.6 and 4.2% of gastric cancers from patients in the United Kingdom, China and Korea (*n* = 961) [203]. A similar rate of 4.1% was found in a smaller study of 267 gastric cancers, which showed conferred sensitivity to FGFR inhibition in gastric cancer cell lines [9]. In 1045 patients with metastatic gastroesophageal cancer, *FGFR2* amplification was identified in 4% of patients [229].

Mutations in *FGFR* are considerably less common than amplification, accounting for 26% of *FGFR* aberrations in cancer. Mutations in *FGFR2* and *FGFR3* were the most common [189]. Of the unique mutations identified for each receptor, all that had been characterized, and the majority that have since been characterized, are known to be activating mutations, and some have known transforming activity. *FGFR2* mutations are most common in endometrioid cancers and gastric cancers [204]. In endometrioid cancers, the majority of *FGFR2* mutations are known to have activating potential [202]. SNPs in the *FGFR2* gene are also associated with increased risk of breast cancer [198].

Fusion proteins arise from chromosomal translocations that result in fusion of two genes. In the case of the FGFR family, the most common fusion proteins include *FGFR3* and *FGFR2* [189]. Fusions involving *FGFR1* and *FGFR4* are rare [230]. Fusions containing FGFR2 are most common in cholangiocarcinoma [199,200,201]. These fusions do not show a preference for a partner as do FGFR3 fusions. The most common *FGFR2* fusions in cholangiocarcinoma are with the BicC family RNA binding protein 1 (BICC1), the sickle tail protein homolog (KIAA1217), S-adenosylhomocysteine hydrolase-like protein 1 (AHCYL1) and the coiled-coil domain containing protein 6 (CCDC6) [199,200]; however, more than 100 other fusion partners have been identified. The biological implications of the majority of *FGFR2* fusions have not been described [230]. *FGFR2-BICC1* and *FGFR2-AHCYL1* fusions have been shown to promote tumorigenesis in vivo, likely resulting in constitutive FGFR2 kinase activation, promoting anchorage-independent growth [231].

### 6.3. FGFR3

*FGFR3* and *FGFR4* amplifications are less common and less well studied than *FGFR1* and *FGFR2* amplifications. *FGFR3* amplification is the most common FGFR amplification event in urothelial/bladder cancer [189,206,207,208].

*FGFR3* mutations are the most common *FGFR* aberration in urothelial cancers and lung squamous cell cancer [189], and are common in cervical and bladder carcinomas. Analysis of 25 *FGFR3* mutations, either identified in cancer or corresponding to common mutations in *FGFR1/2/4,* revealed that the two most common mutations, K650E and N540K, cause large increases in activation of the receptor, whereas less common substitutions at the same sites have less of a pronounced effect on activation. Twelve sites had no notable effect and two resulted in loss of activation. Conflict in some results with previously published works, lead the authors to conclude that SNPs in *FGFRs* may have roles dependent on the cellular context [232].

Fusion of *FGFR3* to transforming acidic coiled coil 3 (TACC3) is the most common *FGFR* fusion protein, which gives rise to a constitutively active FGFR3-TACC3 protein. FGFR3-TACC3 increases FGFR3-dependent signaling [181] and also utilizes TACC3′s role in stabilizing microtubules during chromosome segregation in mitosis [233], which results in aneuploidy prominent in tumors [181]. *FGFR1-TACC1* and *FGFR3-TACC3* fusions are present in 3.1% of glioblastoma multiforme [181], an aggressive malignant primary brain tumor. *FGFR3-TACC3* fusions are also present in other gliomas, urothelial cancers [189,210,234], NSCLC [217,218] and in cervical cancer [215,216].

### 6.4. FGFR4

*FGFR4* amplification is predominantly associated with breast [189,234] and adrenocortical tumors [219], although it is likely that overexpression without amplification occurs more frequently.

A germline *FGFR4* mutation identified in breast cancer cell lines, SNP Glycine (G) 388 to Arginine (R) (G388R), was associated with increased FGFR4 expression. The heterozygous G388/R388 allele and homozygous R388/R388 allele were present at consistent frequencies, 42–49% and 6–11%, respectively, in tumor samples from breast cancer and CRC patients. The homozygous and heterozygous alleles were associated with reduced disease-free survival in both cancer types, as well as lymph node metastasis in CRC [235]. A combined meta and pool analysis of 9354 *FGFR4* G388 allele statuses in multiple cancer types found an association between G388R homo/heterozygosity and reduced overall survival [236]. More recently, a pool analysis of 13,793 cancer patients found the *FGFR4* G388R allele to be associated with increased susceptibility of cancer, which when stratified by cancer type was associated with increased risk of breast and prostate cancer. This SNP was predicted to change protein function [237].

### 6.5. FGFR5

Despite being closely related to the FGFR family, the activity of FGFR5 has been likened more to the CAM Nectin [238,239]. FGFR5 predominantly forms homotrimers at the plasma membrane. However, when co-expressed with FGFR1, it forms heterotrimers (FGFR1:FGFR5:FGFR5) which dimerize downstream of FGF2 stimulation to form a complex of two FGFR1 molecules and four FGFR5 molecules [23]. The constitutive dimers are proposed to function as adhesion molecules, similar to Nectins [239], with no known influence on ERK1/2 signaling or proliferation [238]. *FGFR5* is overexpressed in 10% of CRC cell lines; however, the oncogenic role of FGFR5 has not been characterized yet.

## 7. The Role of Canonical FGFR Cofactors in Cancer

### 7.1. Heparan Sulfate Proteoglycans

HSPGs exist as coreceptors for FGF-FGFR signaling [128]. Additionally, ligand-specificity and subsequent downstream signaling are differentially regulated by the HSGAG side chains attached to the HSPG core domain [240].

The expression of syndecan, a transmembrane HSPG, has been implicated in angiogenesis and lymphangiogenesis in ductal breast carcinoma [241], and in mediating FGFR signaling in breast carcinomas [242]. At the ligand-binding level, syndecans differentially regulate FGFR signaling. Regulating the HS chains of syndecan-1 has been suggested to promote malignancy in premalignant tumor epithelial cells through regulation of FGF binding activity [243]. In lymphoma cells, syndecan-1 can regulate binding of FGF1 and FGF2, but only increases FGF2-dependent signaling [244]. Colocalization of FGF2 in the nucleus and FGFR1 in the perinuclear region of mesenchymal tumor cells is dependent on co-translocation of syndecan-1 [245]. Glypican, a secreted HSPG, has also been implicated in mediating FGFR signaling in tumorigenesis. Glypican-1 is expressed in glioma endothelial blood vessels, but undetectable in healthy brain endothelial blood vessels, and enhanced the mitogenic activity of FGF2-FGFR1-IgIIIc signaling [246].

### 7.2. Klotho

The Klotho family comprises three genes, *KL*, *KLB* and *LCTL*, which give rise to Klotho-α, Klotho-β and Klotho-γ (otherwise known as lactase like or klotho LPH-related protein) isoforms. These genes encode a single-pass transmembrane protein with large extracellular domain, a transmembrane helix and short intracellular C-terminal tail. Cleavage of Klotho family members gives rise to a secreted and soluble version. Klotho in cancer is generally associated with being a tumor suppressor [247].

The role of Klotho-β in mediating FGFR-specific signaling has been mostly studied in the last decade, and has been associated with tumor-suppressor activity in breast cancer cell lines [248] and as a tumor promoter in urothelial carcinoma [168]. Klotho-β has been shown to function as a “zip-code” for recruitment of endocrine FGF ligands to FGFR [249]. Klotho-β is required for increased FGF19-FGFR4 signaling in lung squamous cell carcinoma, to promote cell growth, tumor progression and metastasis, possibly through increased FGF19-FGFR4-mTOR signaling [250]. Klotho-β expression as a coreceptor for FGFR4 has also been implicated in HCC [251], increasing FGF15/19-dependent activity to promote EMT, migration and WNT/β-catenin signaling [252]. This activity has been associated with early tumor reoccurrence [253]. Half of phosphaturic mesenchymal tumors lack FGFR1 fusions; however, Klotho-β overexpression in this subset has suggested that in the absence of FGFR1 fusion, FGFR signaling is still active through Klotho-β [254]. The FGF-FGFR signaling axis has a role in promoting EMT in MM [255]. In MM cells, Klotho-β is expressed alongside elevated FGF23, where an FGF23-Klotho-β-heparanase signaling axis has been implicated in increasing migration and invasion and in metastasis formation [256]. Alternatively, loss of Klotho-β activity has been associated with prostate cancer progression, where decreased expression increases EMT, growth and ERK1/2 signaling [257]. Less established is the role of Klotho-γ in mediating FGFR signaling in cancer. It has been proposed as a biomarker in TNBC, where Klotho-γ is required for survival, and its silencing results in constitutive ERK activation in the absence of a ligand [258].

## 8. Noncanonical Regulators of FGFR Signaling in Cancer

The non-canonical signaling partners of FGFR include extracellular matrux (ECM)-associated, cell adhesion molecules (CAMs), RTKs, other transmembrane proteins and serine/threonine kinases. Such heterotypic interactors and regulators of FGFR signaling have been described in a wide range of cancers for all FGFRs (summarized in Table 3).

### 8.1. ECM-Associated Signaling Partners

FGFR interactions with extracellular matrix (ECM) proteins have been shown to regulate FGFR signaling outputs with possible implications for tumorigenesis. Collagen is laid by tumor cells to form an integral component of the tumor microenvironment, where it regulates processes associated with cancer progression, in particular metastasis [280]. Collagen type IV-mediated upregulation of FGFR1 expression in pancreatic β-cells results in FGFR1-dependent sustained ERK1/2 activation [260]. A more direct interaction has been demonstrated for fibronectin, an ECM glycoprotein largely activated through interactions with integrins. Fibronectin is critical for assembly of the tumor microenvironment, and has known roles in promoting invasion and metastasis, as well as regulating signaling events required for tumor progression [281]. Fibronectin can directly activate FGFR1 by inducing SRC-/integrin-β1-dependent phosphorylation of Y653, Y654 and Y766, promoting an AKT-signaling response over an ERK1/2-signaling response. This, in turn, induces chemotaxis and promotes migration in endothelial cells [282]. More recently, a novel interaction was identified between FGFR1 and galectin-1 (LGALS1)/galectin-3 (LGALS3) in osteosarcoma cells ectopically expressing FGFR1. Galectins are predominantly extracellular glycan binding proteins which are expressed in tumors, with roles in promoting angiogenesis and immune evasion in tumor progression [283]. These interactions are mediated by sugar chains at specific glycosylation sites on the extracellular surface of FGFR1 but result in a galectin-dependent response. LGALS3-FGFR1 interactions altered FGFR1 trafficking, causing FGFR1 to cluster at the plasma membrane and not be internalized. LGALS1 on the other hand activated FGFR1 to elicit more canonical signaling, with a proliferative and survival effect [263]. Given the independent roles of FGFR1 and galectins in cancer, we suggest that galectins could be of relevance when considering targeted therapies regulation of FGFR1 signaling.

Integrins are a family of cell-surface receptors which, in cancer, are predominantly associated with promoting tumor cell migration and invasion through interactions and remodeling of the ECM [284]. Integrin-regulated FGFR signaling has been directly implicated in tumorigenesis, particularly in angiogenesis, a critical step during the formation of metastasis. FGF1-Integrin-αVβ3-FGFR1 crosstalk has been shown to promote angiogenesis and tumorigenesis [265], and later shown to enhance EMT in breast cancer cell lines [266]. Integrin αVβ3 differentially regulates FGF1-dependent signaling to promote changes in cellular outputs, including DNA synthesis, proliferation, chemotaxis and migration [285]. FGFR-integrin crosstalk is not limited to Integrin-αVβ3 in breast cancer. Integrin-β3 is required for FGF2-dependent signal induction in a mouse metastatic breast cancer model, which disrupts FGFR colocalization with epithelial marker E-cadherin in a focal adhesion kinase (FAK)-activation dependent manner to promote EMT [267]. In melanoma cells, an osteopontin-integrin αVβ3 crosstalk has been implicated in mediating FGF2-driven survival [286]. Integrin-β4 signaling in cancer has been associated with promoting tumor progression, and has been suggested as a candidate for immunotherapy in highly metastatic cancers [287]. Integrin-β4 antiapoptotic activity was shown to be down-regulated by FGF2-FGFR1-dependent phosphorylation of Integrin-β4 Y1494 and nuclear translocation [288].

Anosmin-1 (ANOS1, previously the KAL1 gene) is an ECM-associated transmembrane glycoprotein which is able to directly interact with FGFR1 in a complex with HSPG and FGF2 [289]. The ANOS-FGFR signaling axis is often studied in relation to its role in Kallman Syndrome, a developmental disorder predominantly associated with hearing loss and Hypogonadotropic Hypogonadism. The signaling axis differentially regulates ERK1/2 and PI3K signaling downstream of FGFR activation in a cell-type dependent manner [290]. A role for ANOS1-FGFR1 signaling has been indicated by increased expression of ANOS1 in high grade brain tumors when compared to healthy or low-grade brain tumors. In the presence of Integrin β1, ANOS1 promotes motility and invasion in glioblastoma cell lines, which are FGFR inhibition sensitive. ANOS1 activity likely elicits its migratory/invasive activity by activating ECM proteases and modulating cell adhesion [259].

### 8.2. Cell Adhesion Molecules

CAMs have been implicated in promoting migratory properties associated with EMT in cancer. Several CAMs are glycoproteins belonging to the immunoglobulin superfamily (IgSF), one of the largest protein families, which typically have at least one extracellular Ig domain, a single-pass transmembrane domain and a cytoplasmic tail. IgSF CAMs exert their adhesive action through homophilic interactions between IgSF CAMs on opposing cell surfaces, and heterophilic interactions with other cell surface adhesion molecules or proteins on opposing cell surfaces or in the ECM [291]. Neural-CAM (NCAM) can activate FGFR1 independently of FGF-mediated activation, which regulates trafficking and SFK-signaling in a cell type-dependent manner [292]. In pancreatic tumor β-cell neurite outgrowth and adhesion to matrix, NCAM mediated the formation of a complex containing NCAM, FGFR4, FRS2, PLCγ, SRC, cortactin and GAP43. The FGF ligand alone could not promote neurite outgrowth, which was dependent on FGF-integrin-β1 signaling [278]. Recently, an NCAM-FGFR1 signaling axis was identified downstream of transforming growth factor β (TGFβ), that promotes transforming growth factor β (TGFβ)-driven EMT of human proximal tubular epithelial cells [293]. NCAM is also associated with negatively regulating tumor progression. A pituitary tumor-derived FGFR4 (ptdFGFR4) isoform has an alternatively transcribed N-terminal truncated cytoplasmic tail, which can no longer associate with NCAM. NCAM-dependent signaling and adhesion are reduced, generating a ptdFGFR4-mediated invasive phenotype in pituitary neoplasia [279]. A different member of the IgSF, L1CAM, signals through FGFR1 in glioma to promote motility and proliferation [268]. Inhibiting FGFR, integrin or FAK signaling depletes the L1CAM response in L1CAM-positive glioblastoma cells, suggesting an L1CAM-FGFR-integrin-FAK signaling axis is required for L1CAM driven motility and proliferation [269].

Cadherins, a family of transmembrane glycoproteins, form complexes with intracellular membrane-localized catenins to activate signaling pathways and regulate cell-cell contacts, which are implicated in processes involved in tumor initiation and progression [294]. FGFR interacts directly with cadherins (and NCAMs) through the conserved acid-box region in IgIII [295]. The role of FGFR-cadherin interactions is dependent on the type of cadherin, giving rise to a role for FGFR-cadherin signaling as tumorigenic and tumor suppressive. [296,297]. N-cadherin can stabilize FGFR1 at the plasma membrane, preventing ligand-dependent internalization and degradation [270], which in turn enhances a sustained ERK1/2 signal, increases transcription of MMP9 and promotes invasion. In mammary tumor development, the N-cadherin-FGFR-sustained ERK signal has been associated with motility, invasion and metastasis [271]. TGFβ was later shown to increase N-cadherin-dependent expression and signal regulation of FGFR1, which increased AKT signaling and promoted invasion independent of ERK1/2 signaling [298]. FGFR1/4 overexpression in lung cancer has been suggested as a biomarker for poor outcomes and FGFR-targeting therapy when co-expressed with N-cadherin [299]. Similar to N-cadherin, cadherin-11 forms a complex with FGFR and β-catenin to promote neurite outgrowth [300]. Alternatively, FGFR1 can directly interact with N-cadherin at cell-cell contacts to promote cell-adhesion and inhibit migration in the absence of ligand. N-cadherin stimulates FGFR1-dependent activation of SRC and p120 catenin, which, in turn, secures N-cadherin-actin interactions [301]. FGF1 and FGF2 have been show to regulate E-cadherin-Catenin signaling in pancreatic adenocarcinoma [302]. E-cadherin directly regulates FGF-mediated FGFR1 endocytosis into the early endosome, and subsequent nuclear translocation, which is disrupted by stabilizing E-cadherin at the cell surface [303].

Nectin-1, a member of the Nectin family of Ig-like CAMs, is able to bind and signal through FGFR [304]. Although this signaling complex has not been investigated in cancer, Nectin-4 enhancement of HER2 signaling has been investigated in relation to tyrosine kinase inhibitor-resistance in breast cancer [305].

Other adhesion molecules that directly interact with FGFR to mediate signaling in a cancer setting include the focal adhesion nonreceptor protein tyrosine kinase 2 (PYK2). Direct interaction of the PYK2 kinase domain with the FGFR3 juxtamembrane domain links FGFR3 activation to STAT5 signaling in a MM cell line. This interaction reduces MM cell dependence on SFK activation of PYK2 and STAT5 signaling [276].

### 8.3. Other Transmembrane Proteins

Similar expression to FGF (SEF, also IL-17RD) was identified in Zebrafish as a transmembrane protein that directly interacts with FGFR1 and FGFR2 to negatively regulate signaling [306]. SEF, which in the absence of stimulation resides in vesicles in the cytoplasm, colocalizes with FGFR at the plasma membrane to inhibit FGF2-mediated RAS-MAPK signaling [307,308,309]. Interestingly, SEF activity on EGFR is strikingly different. Downstream of EGF, SEF can regulate EGFR trafficking to increase EGFR sorting into the early/recycling endosome, which in the absence of SEF is predominantly sorted into late endosomes [310]. ERK1/2 translocation to the nucleus is also prevented by SEF, which instead promotes cytoplasmic ERK1/2 signaling [311]. Despite these opposing roles in regulating RTK activity, SEF is associated with having tumor suppressor activity. SEF can negatively regulate EMT in breast cancer cells [312] and in prostate cancer [313], in which loss of SEF activity has previously been associated with invasiveness and increased bone metastasis [314]. Similarly, in endometrial adenocarcinoma cells, SEF expression inhibited MAPK activation and proliferation in response to FGF2 stimulation [315].

Polypeptide *N*-acetyl galactosaminyltransferase 14 (GALNT14) belongs to the GALNT family of proteins that initiate O-glycosylation of proteins. GALNT14 is a transmembrane protein with large intracellular domain that has been associated with multiple cancer processes, including the promotion of breast cancer metastasis to the lung. In breast cancer cell lines, GALNT14 O-glycosylation of FGFR1 primes breast cancer cells to respond to FGFR activation [264].

Bone morphogenic protein (BMP) has a role in promoting breast cancer progression, likely in promotion of breast cancer metastasis to the bone [316]. Although not directly linked to cancer, BMP4 has been shown to prime FGFR to response to FGF2, FGF7 and FGF10 activation to increase proliferation in mammary epithelial cells [317].

Neuropilins (NRPs) are nonenzymatic transmembrane glycoproteins with emerging roles in cancer progression, with particular focus on NRP-VEGFR signaling in angiogenesis [318]. NRP1 forms a complex with FGFR1 in the cytoplasm, which increases during EMT in HER2^+^ drug-resistant breast cancer cells. This suggests a role for NRP1-mediated FGFR signaling in breast cancer progression and EMT [272].

### 8.4. Other Receptor Tyrosine Kinases

Although FGFR signaling has been mainly considered to occur as a result of ligand-mediated formation of homodimers, it has been demonstrated that FGFR1-3 can form heterodimers [319] also between alternative splice isoforms [33]. These alternatively spliced isoform heterodimers are proposed to have differential ability to activate signaling pathways. FGFR1-3 heterodimers are evidenced by transphosphorylation between FGFR1 and FGFR2, suggestive of the formation of heterodimers [320]. Del Picollo et al. (2017) showed that two mutations, A391E and G380R, in FGFR3 stabilized the formation of heterodimers between mutated FGFR3 and wild type FGFR1-3. Although not investigated in cancer, heterodimerization within other RTK families, particularly EGFR, is known to mediate critical signaling in tumorigenesis [321].

The juxtamembrane domain of FGFR1-4 is able to directly interact with the tyrosine kinase domain of Ephrin type A receptor 4 (EPHA4) [322], which forms a complex with FRS2α [323]. Further to this, a recent study investigating the strength of RTK heterodimers demonstrated that FGFR1-3 can form a complex with EPHA2, as well as with VEGFR2 [324]. An FGFR1-EPHA4 complex potentiates FGFR-mediated signaling, which when complexed with FGFR2 increases proliferation and migration in a glioma cell line [325]. EPHA4-dependent phosphorylation of a guanine nucleotide exchange factor Rho family GTPase, Ephexin-1, is inhibited by FGFR inhibition, suggesting a coregulatory relationship where FGFR differentially regulates EPHA4 signaling [262]. Similarly, GRB4 (also known as TCK2) interacts with EPHB1 in the presence of activated FGFR1 [326]. EPHA2 expression and activation can be regulated by FGF2 in a colonic adenocarcinoma cell line [327].

FGFR1 has been shown to directly interact with platelet-derived growth factor α (PDGFRA) in response to activation of both receptors, possibly as a mechanism of negatively regulating FGFR signaling [328]. An in-depth investigation into FGFR interactions with PDGFR showed that a direct interaction between FGFR1 and PDGFR-β (PDGFRB) requires the intracellular and extracellular domain of the receptor, as well as FRS2. In response to the PDGFR ligand PDGF-BB, FGFR1 was tyrosine phosphorylated in vascular smooth muscle cells. Even though this complex required FRS2, the proliferation and plasticity response did not depend on activation of ERK1/2 [329]. Although the possible role of FGFR-PDGFR interactions has not been investigated in cancer, it is well established that crosstalk exists between their respective ligands during angiogenesis [330,331].

Fusion proteins containing the RTK rearranged during transfection (RET) have been reported in multiple cancer types. The RET domain of RET-kinesin family member 5B (RET-KIF5B) fusion protein interacts with FGFR and EGFR in endocytic RAB vesicles to increase FGFR and EGFR activation. This RAB-positive endocytic vesicle kinase “signaling hub” contributes to invadopodia formation. Inhibition of FGFR or EGFR, in combination with the RET inhibitor sorafenib, significantly improved response to treatment in human cancer cell lines harboring the RET-HIF5B fusion protein [273]. This highlights the role of differential FGFR trafficking in mediating FGFR interactome and subsequent downstream signaling.

As for other RTK families, interactions and subsequent signaling are yet to be fully understood in cancer. For example, an interaction between FGFR1 and EGFR can marginally increase epidermal growth factor (EGF)-mediated AKT and STAT3 signaling outputs in lung cancer cells [261]. Along this line, we have recently showed that FGFR2-IgIIIb and EGFR engage in reciprocal regulation of each other’s signaling and trafficking in breast cancer cells [275], confirming that FGFR trafficking regulation may become a target for therapeutic intervention.

### 8.5. Serine Threonine Kinases

Complex crosstalk exists between TGFβ and FGFR signaling. TGFβ binds TGFβ receptor (TGFBR). TGFBR1 and TGFRB2 are serine/threonine/tyrosine kinases, whereas TGFBR3 has no intracellular kinase activity. As with all TGFBR signaling, the signaling and cellular outputs downstream of FGFR-TGFBR are context-dependent, heavily dependent on cell type, the environment and other signaling pathways [332]. In hepatoma cells, tumorigenic TGFβ activity is switched to a tumor-suppressive activity when FGFR is inhibited, suggesting crosstalk between FGFR and TGFBR activity promotes TGFβ-mediated malignancy [333]. In tumor endothelial cells, FGF2 switches the TGFβ endothelial-to-myothelial program to differentiate into active fibroblastic cells through activation of E26 transcription factor ELK1, a downstream target of ERK1/2 activation [334]. Alternatively, a negative relationship has been characterized in CAFs, where FGF inhibits TGFβ-promotion of invasion and metastasis [335]. In cancer, a direct interaction between FGFR1 and TGFBR3 has been identified in neuroblastoma cells for which TGFBR3 expression decreases with tumor progression. A complex of FGF2-FGFR1-TGFBR3 activates ERK1/2 signaling and increases expression of inhibitor of DNA binding 1 transcription factor (ID1) expression, ultimately promoting differentiation of neuroblastoma cells. The signaling axis is able to suppress tumor growth and metastasis in vivo [274]. The cell-type-dependent FGFR regulation of TGFβ signaling reinforces the relevance of context dependent FGFR signaling in cancer.

TGFα-activated kinase 1 (TAK1, also MAP3K7) forms a complex with TAK1-binding protein 1 (TAB1) and TAB2/3, and orchestrates a wide range of signal transduction pathways including MAPK, nuclear factor κB (NFκB), noncanonical WNT and AKT signaling, through which it contributes to oncogenic signaling [336]. A direct interaction between FGFR3 and TAK1 increases with mutations in FGFR3 known to occur in MM and bladder cancer. In the presence of these FGFR3 mutations, the interaction likely increases cell adhesion and NFκB-dependent transcription, as demonstrated by the dependency on the expression of TAK1 [277].

## 9. Conclusions and Perspectives

The diversified role of FGFR partners in regulating FGFR activation and signaling supports the relevance of FGF/FGFR pairs in tumorigenesis. By directly interacting with several co-expressed signaling partners, FGFRs can facilitate, amplify or inhibit key tumor progression processes. Not only does this result in signaling events directly dependent on FGF/FGFR, but also indicates a role for FGFR signaling in tumors where *FGFR* genetic alterations are not easily detectable. Rather, FGFR expression can influence activity of alternative oncogenic signaling axes. A large number of studies highlight the context-dependent nature of FGFR signaling, re-emphasizing the importance of the global FGFR proteome and interactome in several cancer types. Comprehensively defining the landscape of FGFR signaling partners in multiple cancers could be exploited to select more efficient drug combinations. As demonstrated by Das and Cagan (2017), RTK signaling is differentially regulated by localization of the receptors, in part due to the colocalized interactome of each receptor [273]. As differential trafficking of FGFR drives intracellular signaling, we speculate that FGFR colocalization with noncanonical signaling partners, including EGFR, may contribute to novel FGFR oncogenic functions. For instance, FGF10/FGFR2-IgIIIb-dependent phosphorylation of EGFR on threonine (T) 693 in breast cancer cells and organoids regulates FGFR2-IgIIIb trafficking and signaling outputs, including cell proliferation [275]. This might have implications in TNBC, where both FGFRs and EGFR are highly expressed [337], and where this phosphorylated site may become a prognostic or predictive marker if a correlation between T693 phosphorylation, clinical parameters, and the response to combined EGFR/FGFR therapies are determined. This idea is supported by the detection of T693 phosphorylation in 50% of the TNBC patient-derived samples analyzed in two independent phosphoproteomics datasets [338,339].

It remains to be determined whether other FGFR signaling cofactors implicated in development may regulate FGFR signaling in cancer. For example, G protein-coupled receptor (GPCR) expression and activity have been implicated in both tumorigenesis and tumor suppression [340]. In addition to this, GPCR-RTK complexes have a role in tumorigenesis [341]. Given that GPCR-mediated activation of FGFR signaling is involved in development [14], it is plausible that the GPCR-FGFR signaling axis plays a role in tumorigenesis yet to be characterized.

In conclusion, canonical FGF-FGFR signaling contributes to tumorigenesis in a context-dependent manner. Noncanonical FGFR signaling partners are critical in regulating oncogenic FGF-FGFR signaling partners and support a role for FGFR signaling in the absence of *FGFR* genetic aberration.

## Figures and Tables

**Table 1:** Examples of cancer types where expression of Fibroblast Growth Factor (FGF) subfamily members regulate oncogenic processes.

FGF Subfamily	Cancer Type	Associated References
**FGF1**	Bladder	[95]
	Breast	[96]
	Melanoma	[97]
	Ovarian	[98]
	Pancreatic ductal adenocarcinoma	[99]
	Small cell lung cancer	[100]
**FGF4**	Bone	[101]
	Gastrointestinal	[102,103]
	Glioma	[104]
**FGF7**	Breast	[42]
	Gastric	[105]
	Non-small cell lung cancer	[106,107]
	Pancreatic ductal adenocarcinoma	[108]
**FGF8**	Colorectal	[109]
	Hepatocellular carcinoma	[110]
	Head and neck	[111]
	Prostate	[112]
	Renal cell carcinoma	[113]
**FGF9**	Colorectal	[114]
	Hepatocellular carcinoma	[115]
	Prostate	[116,117]
**FGF15/19**	Colorectal	[118]
	Endometrial	[119]
	Hepatocellular carcinoma	[120]
	Prostate	[121]
**iFGFs**	Colorectal	[122]
	Prostate	[123,124]
	Triple negative breast cancer	[125]

FGF, fibroblast growth factor; iFGFs, intracellular FGFs.

**Table 3:** A summary of noncanonical interaction partners of FGFR1-4 which have been linked to cancer.

FGFR	Partner	Cancer Subtype	Consequence of Interaction	Associated References
**FGFR1**	Anosmin-1	Brain; Glioblastoma	Promotes motility and invasion in FGFR-dependent manner	[259]
	Collagen type IV	Pancreatic	Sustained ERK1/2 activation	[10,176,177,260]
	EGFR	Lung	Increases AKT and STAT3 signaling	[261]
	EPHA4	Glioma	Potentiates FGFR1-signaling	[262]
	Galectin-1	Osteosarcoma	Activated FGFR1, increased proliferation and survival	[263]
	Galectin-3	Osteosarcoma	FGFR1 plasma membrane clustering	[263]
	GALNT14	Breast	Promotes FGFR activation	[264]
	Integrin-αVβ3	Breast	Increased tumorigensis, angiogenesis and EMT	[265,266]
	Integrin-β3	Breast	Disrupts colocalization with E-cadherin and promotes EMT	[267]
	L1CAM	Glioma	Promote motility and proliferation	[268,269]
	N-cadherin	Breast	Stabilizes FGFR1 at plasma membrane, promoting sustained ERK1/2 signal, increased invasion, motility and metastasis	[270,271]
	NRP1	Breast	FGFR1-NRP1 complex increases during EMT	[272]
	RET-KIF5B, EGFR	RET-KIF5B fusion positive cancers	Increases FGFR activation and promotes inavdopodia formation	[273]
	TGFBR3	Neuroblastoma	Activates ERK1/2 signaling, promoting differentiation, suppressing tumor growth and metastasis	[274]
**FGFR2**	EGFR	Breast	Induces EGFR T693 phosphorylation, recycling and stabilization and increases cell cycle outputs	[275]
	EPHA4	Glioma	Increases proliferation and migration	[262]
	RET-KIF5B, EGFR	RET-KIF5B fusion positive cancers	Increases FGFR activation and promotes inavdopodia formation	[273]
**FGFR3**	PYK2	Multiple myeloma	Increases STAT5 activation	[276]
	TAK1	Multiple myelomaBladder	Cell adhesion and NFκB-dependent transcription	[277]
**FGFR4**	NCAM	Pancreatic	Promote neurite outgrowth	[278]
	NCAM	Pituitary neoplasia	When interaction is inhibited increases invasive phenotype	[279]

EGFR, epidermal growth factor receptor; EMT, epithelial-mesenchymal transition; EPHA4, ephrin type A receptor 4; ERK1/2, extracellular signal-regulated protein kinase 1/2; FGFR, fibroblast growth factor receptor; L1CAM, L1 cell adhesion molecule; GALNT14, polypeptide *N*-acetyl galactosaminyltransferase 14; NFκB, nuclear factor κB; NRP1, neuropilin 1; PYK2, focal adhesion nonreceptor protein tyrosine kinase 2; RET-KIF5B, rearranged during transfection (RET)-kinesin family member 5B (KIF5B); STAT3/5; signal transducer and activator of transcription 3/5; TAK1, transforming growth factor α-activated kinase 1; TGFBR3, transforming growth factor receptor β; T693, threonine 693.

## Abbreviations

aFGF	Acidic FGF
AHCYL1	S-adenosylhomocysteine hydrolase-like protein 1
ANOS1	Anosmin-1 or KAL1
bFGF	Basic FGF
BICC1	BicC family RNA binding protein 1
BMP	Bone morphogenic protein
CAFs	Cancer associated fibroblasts
CAMs	Cell adhesion molecules
CBL	E3-ubiquitin ligase CBL
CCDC6	Coiled-coil domain containing protein 6
CRC	Colorectal cancer
CRK	Chicken tumor virus no. 10 regulator of kinase
CRKL	Closely related CRK-like
DAG	Diacylglycerol
DUSP6	Dual specificity phosphatase 6
ECM	Extracellular matrix
EGF	Epidermal growth factor
EMT	Epithelial-mesenchymal transition
EPHA1/2/4	Ephrin type A receptor 1/2/4
EPHB1	Ephrin type B receptor 1
ER	Endoplasmic reticulum
ER	Estrogen receptor
ERK1/2	Extracellular signal-regulated protein kinase 1/2
ESRP1/2	Epithelial splicing regulatory protein 1/2
FAK	Focal adhesion kinase
FGF	Fibroblast Growth Factor
FGFR	Fibroblast Growth Factor Receptor
FGFRL1	FGFR like 1
FHFs	Fibroblast homologous factors
FRS2	FGFR substrate 2
GALNT14	Polypeptide N-*acetyl* galactosaminyltransferase
GIST	Gastrointestinal stromal tumors
GPCR	G protein-coupled receptors
GRB2/14	Growth factor receptor bound 2/14
HCC	Hepatocellular carcinoma
HER2	Human epidermal growth factor receptor 2
HSPGs	Heparan Sulfate Proteoglycans
ID1	Inhibitor of DNA binding 1 transcription factor
iFGF	Intracellular FGF
IgI-IgIII	Immunoglobulin-like looped domains I-III
IgSF	Immunoglobulin superfamily
IP_3_	Inositol trisphosphate
KGF	Keratinocyte growth factor
KIAA1217	Sickle tail protein homolog
KIF5B	Kinesin family member 5B
LGALS1/3	Galectin-1/3
MAPK	Mitogen activated protein kinase
miRNA	Micro RNA
MM	Multiple myeloma
MMP9	Matrix metallopeptidase 9
mTOR	Mammalian target of rapamycin
NCAM	Neural cell adhesion molecule
NFκB	Nuclear factor kappa-light-chain-enhancer of activated B cells
NRPs	Neuropilins
NSCLC	Nonsmall cell lung cancer
PDAC	Pancreatic ductal adenocarcinoma
PDGFRA	Platelet derived growth factor α
PDPK1	3-phosphoinositide-dependent protein kinase 1
PI3K	Phosphatidylinositide 3-kinase
PI3KR1/2	PI3K subunit alpha/beta or p85 PI3K subunit
PIP_2_	Phosphatidylinositol 4,5-bisphosphate
PIP_3_	Phosphatidylinositol 3,4,5-trisphosphate
PKC	Protein kinase C
PLCγ	Phospholipase C gamma
PP2A	Protein phosphatase 2A
PTPRG	Protein tyrosine phosphatase 6
ptdFGFR4	Pituitary-derived FGFR4
PTPN11	Protein tyrosine phosphatase nonreceptor type 11
PYK2	Nonreceptor protein tyrosine kinase 2
RET	Rearranged during transfection
RSK2	Ribosomal S6 kinase 2
RTK	Receptor Tyrosine Kinase
SEF	Similar expression to FGF
SFK	SRC-family kinases
SH2	SRC homology 2
SH3BP4	SH3-binding protein 4
SHC	SRC-homology-2 containing transforming protein
SHIP2	Phosphatidylinositol 3,4,5-trisphosphate 5-phosphatase 2
SNP	Single nucleotide polymorphism
SPRY1/2/4	Sprouty 1/2/4
STAT1/3/5	Signal transducer and activator of transcription 1/3/5
TAB1	TAK1-binding protein
TACC1/3	Transforming acidic coiled coil 1/3
TAK1	TGFα-activated kinase 1
TGFBR1/3	TGFβ receptor 1/3
TGFβ	Transforming growth factor beta
TNBC	Triple negative breast cancer
VEGFR	Vascular endothelial growth factor receptor
Y	Tyrosine
